# Aphrophoridae Role in *Xylella fastidiosa* subsp. *pauca* ST53 Invasion in Southern Italy

**DOI:** 10.3390/pathogens10081035

**Published:** 2021-08-16

**Authors:** Ugo Picciotti, Nada Lahbib, Valdete Sefa, Francesco Porcelli, Francesca Garganese

**Affiliations:** 1 Dipartimento di Scienze del Suolo, della Pianta e degli Alimenti, University of Bari Aldo Moro, 70126 Bari, Italy; ugo.picciotti@uniba.it (U.P.); nadalahbib48@gmail.com (N.L.); valdetesefa@gmail.com (V.S.); francesca.garganese@uniba.it (F.G.); 2 Department of Marine Science and Applied Biology, Laboratory of Plant Pathology, University of Alicante, 03080 Alicante, Spain; 3 Faculty of Sciences of Tunis, University of Tunis El-Manar, Tunis 1068, Tunisia; 4 INRAT—National Institute of Agronomic Research of Tunisia, Laboratory of Plant Protection, Rue Hédi Karray, Ariana 2049, Tunisia; 5 CIHEAM—Centre International de Hautes Etudes Agronomiques Méditerranéennes, Mediterranean Agronomic Institute of Bari, 70010 Valenzano, BA, Italy

**Keywords:** bacterial diseases of non-wood plants, environmental IPM, alien, invasive, quarantine

## Abstract

The *Philaenus spumarius* L. (Hemiptera Aphrophoridae) is a xylem-sap feeder vector that acquires *Xylella fastidiosa* subsp. *pauca* ST53 during feeding on infected plants. The bacterium is the plant pathogen responsible for olive quick decline syndrome that has decimated olive trees in Southern Italy. Damage originates mainly from the insect vector attitude that multiplies the pathogen potentialities propagating Xf in time and space. The principal action to manage insect-borne pathogens and to contain the disease spread consists in vector and transmission control. The analysis of an innovative and sustainable integrated pest management quantitative strategy that targets the vector and the infection by combining chemical and physical control means demonstrates that it is possible to stop the *Xylella* invasion. This review updates the available topics addressing vectors’ identification, bionomics, infection management, and induced disease by *Xylella* invasion to discuss major available tools to mitigate the damage consequent to the disease.

## 1. The Insect-Borne Plant Pathogen

*Xylella fastidiosa* Wells et al., 1987 (Xf) [1] is a xylem-restricted “fastidious” bacterium that lives in plant xylem and foregut vector lumina [2,3,4] of some xylem-feeders auchenorrhynchan [5].

Some phytopathological characteristics related to Xf (e.g., diversified range of plant hosts) are due to the ability of the bacterium to acquire DNA from the environment through horizontal transfer [6]. Xf is able to infect more than 300 different host species including crops, ornamental and spontaneous plants [7,8].

In addition, *Xylella* strains can recombine among themselves [9,10], enriching the gene pool. The genetic diversity of Xf strains allows them to infect several plants species but rarely diseasing them lethally. The bacterium may play a role in plant health, causing nonspecific water shortage symptoms or damage by plugging the xylem vessels [4,11,12,13,14]. Moreover, Xf uses part of its vector’s cuticle as a nitrogen source employing enzymatic chitinase to dissolve exoskeleton [15]; the vector also ingests xylem sap [16] for nitrogen and carbon sources.

*Xylella fastidiosa* is an exotic pathogen introduced in Europe by the trade of asymptomatic coffee plants from Costa Rica [17]. Schaad et al. [18] proposed three Xf subspecies: *Xylella fastidiosa* subsp. *fastidiosa*, previously named *Xylella fastidiosa* subsp. *piercei* (type strain ATCC 35879T, causing grape Pierce’s disease); *Xylella fastidiosa* subsp. *multiplex* (type strain ATCC 35871T, causing plum leaf scald disease/phony peach disease); *Xylella fastidiosa* subsp. *pauca* (type strain ICPM 15198, causing citrus variegated chlorosis or CVC disease). Furthermore, literature reports *Xylella fastidiosa* subsp. *sandyi* (type strain ATCC 700598, causing oleander leaf scorch) [19,20] and *Xylella fastidiosa* subsp. *morus* (found on *Morus alba* L.) [21]. The *Xylella* subspecies, namely *sandyi* and *morus*, were recently included in *Xylella fastidiosa* subsp. *fastidiosa* [22].

Moreover, Su et al. [23] described a new *Xylella* species on *Pyrus pyrifolia* (Burm.f.) Nakai, 1926, in Taiwan named *Xylella taiwanensis* Su et al., 2016.

**Figure 1:**
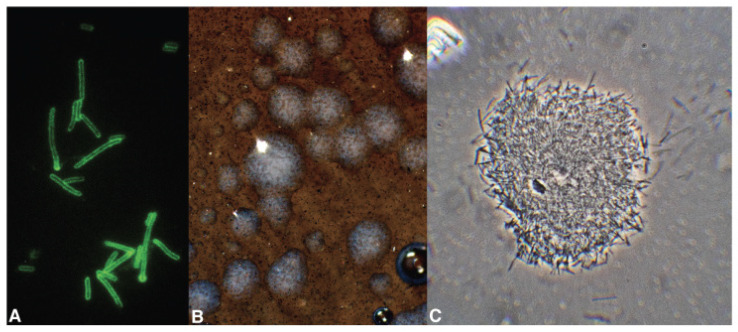
(**A**) Immunofluorescence-positive *Xylella* cells; (**B**) colonies of *X. fastidiosa pauca* ST53 isolated from a spittlebug-infected periwinkle on epi- and transilluminated BCYE medium; (**C**) *X. fastidiosa pauca* ST53 from BCYE colony seen under a phase-contrast compound microscope.

The olive strain found in 2013 in the Gallipoli area (Apulia, South Italy) that cause the Olive Quick Decline Syndrome (OQDS or CoDiRO, in Italian) is *Xylella fastidiosa* subsp. *pauca* ST53 (Xfp53) [24,25], a compact phylotype constituted of some Costa Rican strains (Figure 1). Xfp53 appears different from the other *pauca* strains isolated in Central and South America on *Citrus sinensis* (L.) Osbeck, 1765 and OQDS strain can infect *Nerium oleander* L., 1753, *Coffea arabica* L., 1753 and *Mangifera indica* L., 1753 [26,27], as well as *Olea europaea* L., 1753. Other *X. fastidiosa* subsp. *pauca* strains infecting olive trees have also detected in Argentina [28] and Brazil. The latter strain differs from the Xfp53 strain basing on MLST analysis [29].

*Xylella fastidiosa* is in the quarantine organism list [30,31] and it is known as a biological weapon for the damage it can infer to a country’s crop production system [32,33,34,35,36,37]. The bacterium was detected in Europe in 2013 [38] and could have suspiciously entered Kosovo (wrongly mentioned as Slovenia in Janse 2006) [39,40].

*Xylella* moves from plant to plant and invades the territory [41,42] mainly by insect vectors. Some Aphrophoridae can also acquire the pathogen from infected plants to transmit it to other plants [43,44]. Aphrophoridae-borne plant pathogen transmission could be interspecific or intraspecific. *Xylella fastidiosa* may spread solely by vectors, and Xf invasion is vector-mediated, eventually. Vector–host–pathogen interactions determine whether or not an incursion or isolated pathogen outbreak will lead to settlement, persistence, and resulting epidemic development [45].

As shall be in most cases of vector-borne pathogens, the vector management is the primary pathogen management tool [46,47,48,49,50,51]. *Xylella* has secondary ways to pass from infected to free host plant apart from the vectors, e.g., grafting [52,53] or interplant self-grafting (inosculation), but secondary spreading means are negligible, and infected plant propagation is a way to invade a cultivated territory.

## 2. Non-Vector/Vector Pest Damage

The interest in vector-borne pathogen and vector control management rises because the interaction among the actors—vector, pathogen, and crop—causes relevant damage. We consider here first the proportionality of the damage inflicted by the insect pest alone, then discuss the damage due to the vector–pathogen interaction. The damage is more relevant in a vector–pathogen interplay than the damage eventually due to the Aphrophoridae or the Xf alone.

### 2.1. Non-Vector Pest Damage

Usually, pest damage is directly proportional to single insect actions, where every single action is not restricted to a particular place or a limited period. Overall, the damage is limited because space and time hinder the pest population as its dispersal ability and lifespan. Usually, a single insect can feed on a specific part of its host/food plant (e.g., *Bactrocera oleae* (Rossi, 1790) or *Prays oleae* Bernard, 1788) [54,55] or cause damage by laying the eggs into a plant organ (e.g., Cicadidae) [56]. In non-vector pest, the probing (=an unconcluded feeding attempt) is not genuinely damaging or not at all. A single pest individual is hardly lethal for the plant it targets, eventually ruining a particular organ. Often, a single pest individual may damage a part of the total production for a perennial horticultural crop in a particularly productive year [57,58,59,60]. Most of the pest damage appears as inflicted at the end of a limited interval from the pest action. The discussion may consider if the crop is annual or perennial to quantify the damage amount properly.

However, the pest number/damage proportionality allows using such proper techniques as pest trapping and similar approaches for thresholds evaluation [61]. In control actions, the damage forecast should promptly compare the damage value with the control cost [62,63]. The pest control issue consists of managing the pest population to get acceptable damage without suppressing all the pest individuals.

### 2.2. Vector Pest Damage

A vector sums its proper direct damaging ability over the infested plant with the additive damage inflicted by the borne pathogen. The damage due to the pathogen transmission is connected with direct pest behaviors, e.g., feeding or egg laying, and indirect conduct, such as plant probing [64]. The vector could spread the pathogen among susceptible plants in both cases [65]. Generally, vector–pathogen transmission can be non-persistent, semi-persistent or persistent [65]. Non-persistent pathogen transmission occurs within minutes from pathogen acquisition, and retention occurs on insect stylets [66]. The vector can rapidly lose all the borne pathogens in this transmission path, and multiple encounters with infected host plants are required for the vectors to remain viruliferous [66]. Semi-persistent pathogen retention can last for days, and the pathogen thrives in the insects’ alimentary canal [67]. Pathogen semi-persistent transmission occurs after hours or days of feeding to get the microorganism. Finally, vectors are infectious until death after a single encounter with an infected plant by persistent transmission. It takes hours or days for vectors to acquire the persistent pathogen. In Xf, the acquisition consists of one event, as fast as the non-persistent modality, but with persistent propagative, non-circulative modality. Adults Aphrophoridae have a persistent relationship with Xf [43,68].

Vector pest damage is greater than the sum of single insect actions, because every single inoculation projects the borne pathogen and the consequent damage in space and time. An infection on a plant organ can propagate to the entire plant [69], depending on the infected plant size. In time, the pathogen can continue to inflict damage after the vector death for years [70].

Therefore, it is necessary to consider the plant habit and damage severity. A lethal infection and consequent incidence of disease in trees orchards will substantially causes the annihilation of all future production, struggling the agricultural production system [71]. Vector-bearing poses a severe risk of preventing any other plant production for the crop if subjected to the pathogen spreading.

Vector damage can be lethal by itself to the plant if the pathogen is lethal, making damage essential and not allowing the use of techniques for threshold assessment. Conventional approaches do not have functional timing or resolving power to avoid the first transmission of the pathogen (damaging event) [72,73] preventing the infection and pathogen-driven escalation.

#### 2.2.1. Vector Species Identification

Vectors responsible for Xfp53 spreading are all indigenous and xylem-feeders, as expected for Xf transmission [71,74,75]. Phloem-feeders could acquire Xf, but they miss the ability to transmit it [76]. Therefore, xylem-feeders may occasionally acquire non Xf-pathogen from phloem vessels during food probing [77]. The relationship between the Xf and the vectors is persistent and propagative [68,78] but restricted only in the Aphrophoridae adult stage, where Xf behave as a non-mutualistic ectosymbiont.

Italian vectors are all Aphrophoridae, and the most apparent Mediterranean vectors shall pertain to the same family. The primary vector is *Philaenus spumarius* (L., 1758, the meadow spittlebug), while less efficient vectors are *Neophilaenus campestris* (Fallén, 1805) and *Philaenus italosignus* (Drosopoulos and Remane, 2000) [79].

*Philaenus spumarius* (Ps) is the most diffused and common spittlebugs species in the Palearctic area [80,81] with continental climate, ranging from Portugal to Primorye Russian Territories (Πpимopcкий кpaй = Primorskij Kraj). Furthermore, Ps was introduced and acclimated in the Nearctic area with subtropical, tropical, temperate, and arctic climate, where it can transmit Xf on grape [82].

**Figure 2:**
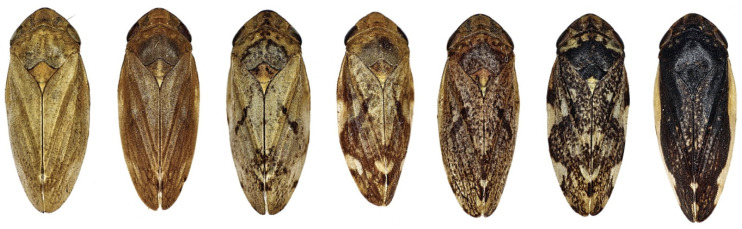
Some color/pattern morphs of *Philaenus spumarius* from dorsum.

Features as inherited polychromies [83,84,85,86] (Figure 2), egg overwintering, cold-interrupted pre-reproductive parapause [87,88], winter/early spring juvenile development, adult’s ethological preference for fresh and shaded growing dicot plants, suggest that Ps originates in the Palearctic area [86,89]. Climate change affects the distribution of Ps because it has little ability to adapt bionomically or ecologically to rapid environmental conditions changing [90].

In the Mediterranean basin, further seven *Philaenus* species apart Ps exist, namely:

– *Philaenus loukasi* Drosopoulos and Asche, 1991 (Greece) [81,91];
– *Philaenus arslani* Abdul-Nour and Lahoud, 1996 (Lebanon) [92];
– *Philaenus tesselatus* Melichar, 1889 (Tunisia) [93,94];
– *Philaenus signatus* Melichar, 1896 (Greece) [81];
– *Philaenus italosignus* (Italy) (Drosopoulos and Remane, 2000) [81];
– *Philaenus maghresignus* Drosopoulos and Remane, 2000 (Morocco, Algeria, Spain and Tunisia) [81,95];
– *Philaenus tarifa* Remane and Drosopoulos, 2001 (Iberian Peninsula) [96].

Apart from Ps, the other *Philaenus* species have a Central-South Mediterranean distribution [97]. Only *P. signatus* goes Northern enough to approach European continental areas (Balkan peninsula). However, with plenty of olive and citrus orchards, the East Adriatic shores belong to the *Csa* group (hot summer Mediterranean climate) and the continental Balkan peninsula areas to *Csb* (warm summer Mediterranean climate) and to *Dfb* (warm summer humid continental climate) according to the Köppen-Geiger system [98].

Available data from the male’ genitalia study [94] suggest speciation occurred (or is ongoing) [86] from a plesiomorphic ancestor of the actual Ps originating apomorphic taxa in new territories and shifting on monocot host plants. The last short-glaciation—the Younger Dryas (from 12,900 to 11,700 years ago)—and the subsequent warming [99] provoked the consequent sea level rise and fall that interplayed with territories, flora, and species dispersion to originate actual *Philaenus* complex biogeography.

This biogeographic [100] interpretation also includes the host plant shift from continental central European cold-intermediate to hot-dry Mediterranean environments. South Aprhophoridae’s host range includes various herbs and spontaneous plants usually ignored by Ps pre-imaginal instars as the genus *Asphodelus* [101] and *Eryngium* [102] or trees and shrubs [95] during the adult stage.

Estimates indicate that the evolutionary history of Ps is most likely related to climate changes of the Pleistocene epoch (2.588–0.0117 MY ago) [103,104].

#### 2.2.2. Morphology and Identification

**Figure 3:**
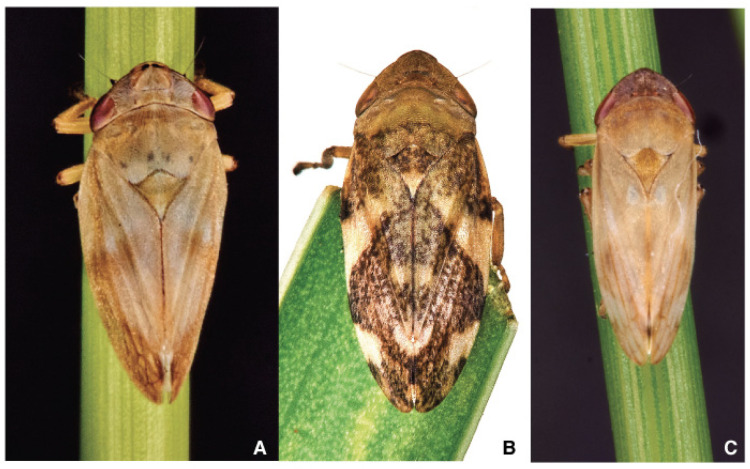
Adults of (**A**) *Philaenus spumarius*; (**B**) *Philaenus italosignus*; and (**C**) *Neophilaenus campestris*.

*Philaenusspumarius* (Figure 3A) adult head is broad, short, and equal in width to the rounded pronotum [105]. The vertex is angular and bluntly and twice as wide as it is long. Eyes are prominent on the side of the head. Ocelli are as far apart from each other as they are from the eyes. The antennae insert the genae between the eyes [106,107]. In Ps, the antennae are in the transition zone between the fronto-clypeus and the compound eyes. The antennae are inserted almost perpendicularly on the cuticular wall through an articulated socket, resulting in antennomere oriented towards the sides of the insect’s body [106]. The antenna is about 820 mm long in both male and female, with three segments: a short cone-shaped scape (length about 140 mm) connecting the antenna with the head capsule, a cylindrical pedicel (length about 120 mm) and a long thread-like flagellum (length about 750 mm) [106].

**Figure 4:**
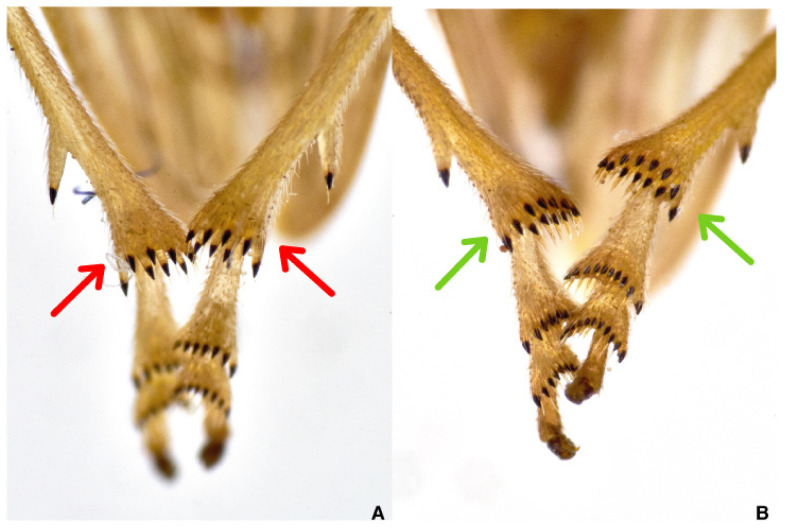
Spurs number on Aphrophoridae hind tibiae: (**A**) *Philaenus* with eight spurs (red arrows); (**B**) *Neophilaenus* with twelve spurs (green arrows).

The general body shape is squat and stout, not much pubescent, giving the insect a frog-like appearance because they expose the posterior legs. The labium does not extend beyond the middle coxae [108], and the hind tibiae have two stout spines each and a crown of eight smaller spurs near the tibio-tarsal joint [109] (Figure 4A).

Tegmina (moderately sclerotized forewings, *sensu* [110]) rest tent-shaped over the abdomen; it is convex on the costal margins and bluntly rounded, meeting beyond the tip of the abdomen. The apex of the tegmina is not reticulated [108].

The apex of the clavus is acute, and the corium is without a terminal membrane. The Ps adults measure from 5.5 to 6.0 mm long and range from 2 to 2.5 mm wide [108]. Adult color forms dorsum vary from pale straw to nearly black. Tegmina show various marks, spots, oblique cross bands, or longitudinal stripes assemblages because of the different morphs’ appearance.

*Philaenusitalosignus* (Figure 3B) is distinguishable from other species by studying slide-mounted genitalia [81]. In the case of genus *Philaenus*, the size of adults is not so affordable character status to identify individuals from a previously not studied population. The suggestion is to identify male slide-mounted genitalia to identify *Philaenus* species.

*Neophilaenuscampestris* (Figure 3C) differs at first sight from Ps because the costal margins of tegmina are straight at rest, and a crown of 12 smaller tibial spurs [111] (Figure 4B) are about the tibial-pretarsus joint. *N. campestris* [112] appears slender in comparison with genus *Philaenus* representatives.

**Figure 5:**
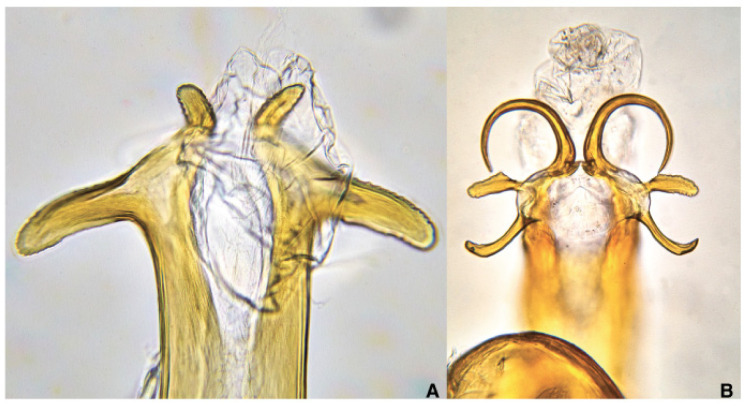
Aedeagal tips of (**A**) *Philaenus italosignus* and of (**B**) *Philaenus spumarius*.

Examination of male genitalia—aedeagus tip—from the posterior end of the abdomen allows the discrimination of the two species of *Philaenus*: *spumarius* and *italosignus*. The aedeagus tip of *P. italosignus* shows two apparent pairs of straight processes (Figure 5A) [81]; however, three pairs of processes appear at the aedeagus tip of Ps (Figure 5B). The process orientations also matter, but the shape of aedeagal processes permits the discrimination between these two taxa.

Eggs are elongate oval, broadly rounded at one end and slightly tapered at the other. They are about one mm long and a third as comprehensive, approximately. Females prefer to lay the eggs on monocot remains [89,113] as dry culm-embracing leaf sheets. *Sorghum halepense* (L.) Pers. often hosts egg batches in South Italian orchards.

**Figure 6:**
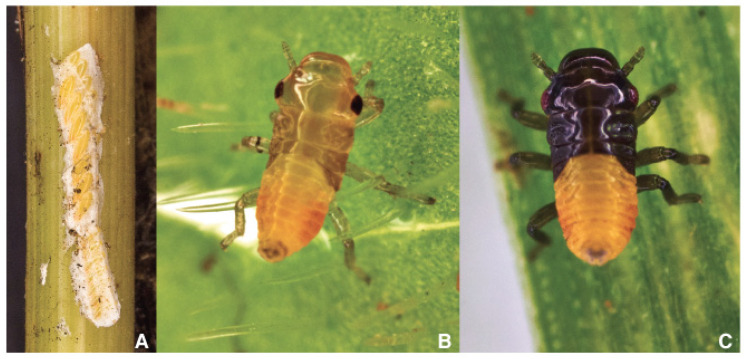
(**A**) *Philaenus spumarius* superimposed egg-batches, (**B**) *Philaenus spumarius;* and (**C**) *Neophilaenus campestris* newborn naiads.

A hardened frothy whitish matter (cement) glues the eggs in series of 2–30 elements, also superimposed (Figure 6A). The observer may guess the presence of the eggs scrutinizing for cement remain exceeding the edge of the sheath. In experiments with caged spittlebugs, Weaver and King [108] reported 17 eggs per female, Cecil [114] obtained 16 eggs per female, and Mundinger [115] refers that the number ranged from 18–51. Weaver and King [108] also found considerable variation in the number of eggs deposited on different materials, straw is preferred [116].

Freshly laid eggs are pale-yellow, passing to a deeper orange-yellow with a dark-orange/red spot that appears before overwintering. During development, a hatching membrane in the form of a smooth tightly stretched pellicle coats the body, and after the hatching, the membrane keeps into the chorion near the newborn exit [117].

*Philaenus spumarius* can produce more than one egg from each ovariole [118] and the number laid depends mainly on the length of the season. Therefore, the overwintering egg density is not directly related to the density of adult females that introduces a strong trigger into the population dynamics forecast [113].

Juvenile development consists of five instars: three naiades plus two nymphs. Newborn Ps naiades (Figure 6B) are orange with a sclerotized prosoma (head and thorax) changing through yellow to green nymphs by aging. *N. campestris* juveniles (Figure 6C) have almost black prosoma (head + abdomen) and yellow abdomen; the color scheme does not change with the growth.

Each juvenile instar has a different head size helping age identification by measuring the head capsules width that is sclerotized and less prone to size changes during the intermolt. Other morphological changes occur, apart from the absolute increase in head capsule size. More or less than proportional changes occur at molting: legs length increases, tarsi are less bulbous, and the abdomen flattens dorso-ventrally. The length of the body increases with growth [119]; however, it is unreliable for the considerable variability among individuals of the same instar due to, e.g., engorgement, or because the abdomen extends and retracts continuously in the same individual, or for differences due to preservation techniques.

**Figure 7:**
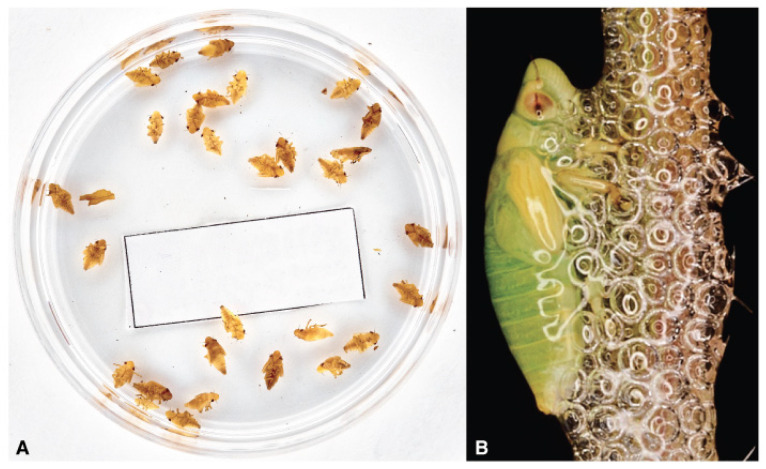
(**A**) Field-collected juvenile sample of *Philaenus spumarius’* 3rd, 4th, and 5th instars; (**B**) *Philaenus spumarius’* 5th instar nymph showing wing buds venation.

Today, discriminating between naiades and nymphs is almost negligible in heteropteran juvenile description, even if morphological differences are often noticeable. The presence of nymphs in the field population of spittlebugs alerts for the timing vector control actions [120]. For this, we suggest discriminating naiades from the nymphs on the base of the two pairs of prominent wing buds (Figure 7A) and last nymph because of apparent venation in the buds (Figure 7B). Wing buds are evident in field observations and alert the operator to execute the control actions versus juveniles timely [72].

Accurate observations show that the spittlebugs juveniles’ cuticle is hydrophilic and fully wettable. Contrary to the majority of land-living insects, the young spittlebugs share with water-living juvenile (e.g., Odonata) or larvae (e.g. Culicidae) a hydrophilic cuticle permitting their life in liquid [121].

## 3. Vector Bionomics

**Figure 8:**
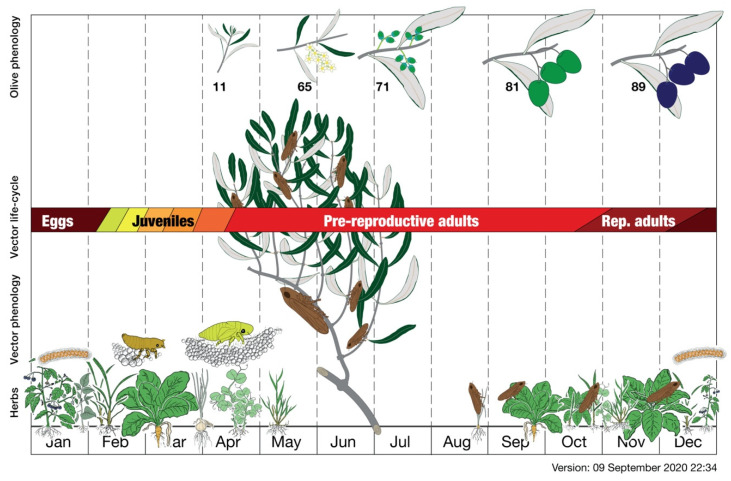
*Philaenus spumarius* life cycle related to herbs and olive trees phenology [127] (drawing from [73]).

The life history of Ps is based on a relatively long egg overwintering, a short winter-early spring pre-imaginal development, and a relatively long adult life interval from spring to winter [84,122,123,124]. The life cycle ends with the female dies after laying eggs (Figure 8) [108].

Ps feeds on a vast repertoire of host plants in the field [125], either herbs, or bushes or trees. *Philaenus spumarius* participates to that 10% of phytophagous insects that feed on more than three different plant families [126]. Ps prefers Asteraceae, Fabaceae, Apiaceae, and Lamiaceae as food plant, both in juvenile and adult instars [7].

The egg is the resistance stage for the insect [85]. Most of the eggs are laid near the ground in slits or similar receptacles, offering two facing surfaces [128]. Urged to lay eggs, in rearing, the female accepts plastic foils, cardboards, and similar substrates to oviposit as the internal wall surfaces or cotton wool of plastic rearing boxes.

The temperature modulates the hatching [88,129] that needs exposure to a long period below 5 °C to break the eggs diapause. In Southern Italy (Apulia region), first, newborns appeared vagrant in middle February [130], but the peak in hatching is unclear, maybe because the weather varies considerably. Hatching lasts about one month, and different first instar naiades may coexist in the field for an extended period because of a long hatching interval.

**Figure 9:**
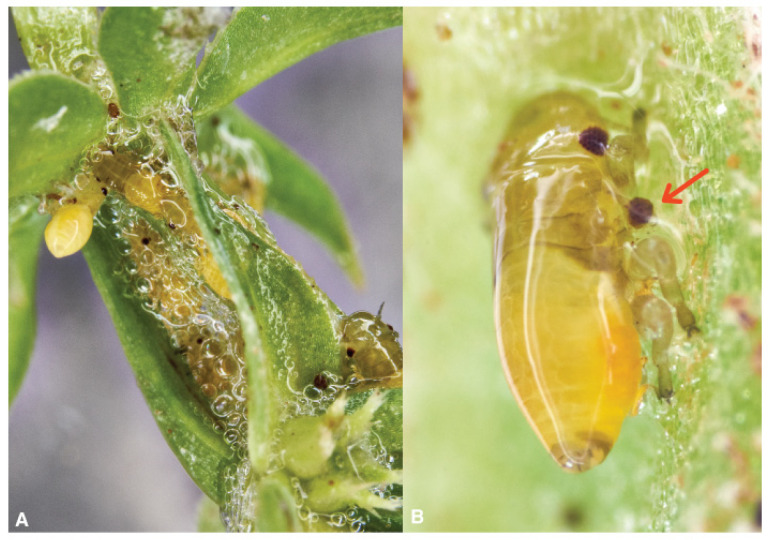
(**A**) Juveniles *Philaenus spumarius*’ naiades aggregation on *Lotus* sp.; (**B**) detail of a 2nd *Philaenus spumarius*’ instar naiad starting bubbling just after the first molt (red arrow on the eye from 1st instar exuvia).

After hatching, the first naiades must make their way from abandoned choria to a suitable host plant. Vagrant first naiades are perhaps the most delicate since nymphs’ subsequent steps have froth to shelter helping survivorship [85,123]. Therefore, the first naiades are likely found on plants with basal leaves rosette or offering closely apposed leaf and stem surfaces [108,131]. Most olive groves host a relevant spontaneous herb layer during egg hatching time, and the young naiades may aggregate on preferred plants. The juveniles will crawl up to find a sheltered site on a suitable succulent plant to insert the stylets [115,116]. Feeding elicit the secretion/excretion of mucus and faeces, and a small liquid pond expands centered on the first instar. Despite the efforts, the very young insects cannot produce true spittle but a mix of liquid and some scattered bubbles, even if several individuals aggregate (Figure 9A) [132]. Foamy mass does not adequately shape the fluid prevalence that stays apart by gravity, and the insect bodies are too small to maintain around the bubbles. The second instar produces more fluid and feces and the insect starts bubbling into the liquid to form the spittle [85,133,134], the sequence repeats each molt (Figure 9B). The juveniles are spittle-protected from drying, T °C peaks, sun, and other environmental stresses [108,131,135,136,137,138,139,140]. The spittle is a truly liquid niche for spittlebug juveniles that catch occasional visitors. The spittle could be involved in juveniles Aphrophoridae breathing [141].

Naiades’ and nymphs’ ensembles are ordinary in field condition and represent adaptive behavior [132] and several juvenile individuals on the same host plant can thrive in shared bubble masses [85].

Older naiades and nymphs set on the plants lonely, preferably head down and abdomen up. Later, mucus and bubbles slip on the body and in between the plant and body space. Spittlebug extends the abdomen over the spittle mass and draws air into a groove on the ventral surface of its abdomen [142]. Overlapping pleural prolongations form the sternal groove with the abdominal spiracles [133,141]. The insect rises the anus out of the spittle and retracts the abdomen bubbling the air into the fluid, several times per minute. The secretion of the Batelli glands [133,143,144,145] seems to ameliorate the bubbling in the anal fluid sheltering under a foamy cover.

The naiades (the first three juvenile instars) and nymphs (the last two instars) produce 1–3 cm long spittle masses on a high number of annuals or perennials herbs, shrubs, and trees host plants [146,147]. The juveniles pierce the plant tissue with their stylets and suck the xylem plant sap they feed [148]. The plant xylem sap exceeds in water, the excess fluid passes through the alimentary canal, and the anus eliminates it [116,141].

Juveniles last from 35 to 58 days in the field [108]. This period is degree-day triggered so that the juveniles’ lifespan may last 30 days only or extend to 110 days, manipulating the temperature [149,150]. Juveniles are abundant in spittle on herbs either in natural, or agricultural habitats [151] or urban environments [73] but are not as significant vectors because they cannot move over distances.

**Figure 10:**
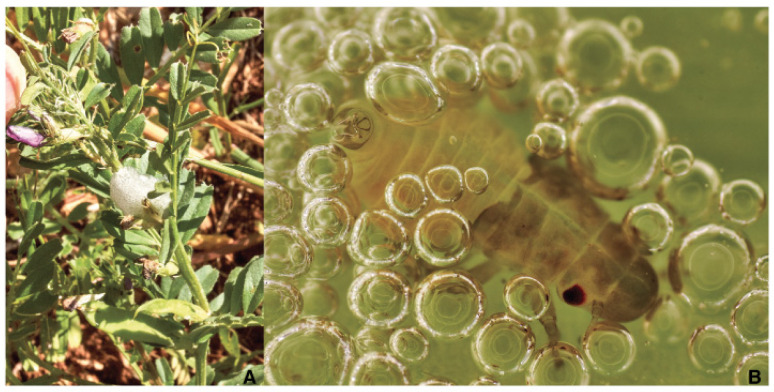
(**A**) Foamy mass on *Vicia* sp.; (**B**) *Philaenus spumarius* 3rd naiad instar bubbling on host plant.

Naiades and nymphs spend their life feeding and managing their position into the herb layer (Figure 10A). They are not immotile, but they change the feeding place on the same plant or change the host plant with an adjacent one [147]. Most of the position changes consist of moving near to the plant tip and in a more exposed position as the younger ages. After about seven weeks from the hatching, the eclosion occurs on the herbs also. The last nymph stops bubbling, and the fluid within the foam drops from the spittle, drying it in the ending the pre-imaginal life [116]. A chamber opens in the foam around the nymph that molts to adult [141]. A neat cavity stays in the spittle shortly after Ps eclosion and its abandonment.

The individuals will become infective as adults when they move from herbs to the olive trees (or other reservoir plants) during the spring–summer and after acquiring Xf from infected plants [152]. Adults are free-living insects and can fly, but they prefer crawling or jumping [34,103,153,154]; in fact, flying seems more a gliding than an active fly. Adults feed on any available food plant and excrete liquid feces, projecting droplets around with any bubble inclusion.

The adults usually remain in the area until the food plants stay available and before herbs dry out [155]. As available food plants run out the adults mass-move to another available food source around, even to gymnosperms [156,157,158] as *Pinus* spp., *Cupressus* spp., or *Tuja* spp. There is a sort of summertime horizontal adults’ circulation over the country that pushes adults to abandon the areas of juvenile lifetime because herbs dry after flowering and hydric stress makes not available hardened woody plants. Adults will re-enter in the orchards after the first rains approximately in late August searching for the spontaneous perennial herbs (Hemicryptophytes and Cryptophytes) re-sprouting [36,72,73,155,159].

Adults mate continuously throughout the summer, and eggs appear in ovaries in August, but females refrain from oviposition because of extended daylight and the high temperature that induces a parapause [87,160,161]. Oviposition starts in September, and eggs undergo overwintering diapause [85,158]. Egg-laying continues until the death of the last females in December [162]. Males start to die before the females; all insects die once exhausted they role and field population declines quickly in synchrony by climate, like other animal populations [163,164].

## 4. Damage

In OQDS species of Aphrophoridae, juveniles are not harmful but adults inflict their most serious damage [73]. However, the attitude to damage plants is contextual and not intrinsic to insect action. Spittlebugs originate essential damage to olive plants and orchards because of their ability to transmit Xfp53. Alternative damage by host plant or food plant feeding is due because Ps can ingest xylem sap 150–200 times the body weight in a day (more than 10 times the ratio typical of the phloem-feeding pests) [165,166].

Adult belonging to few species of Aphrophoridae can transmit Xfp53 [167] and further strains of the bacterium on their food plants. *P. spumarius*, *P. italosignus*, and *N. campestris* demonstrated to inject Xfp53 into the xylem vessels through Apulian olive orchards, inducing extensive OQDS and damaging olive trees to death because of sudden and massive dieback [168] with different symptoms intensity on plant and in the time.

### Damage by Xylella fastidiosa pauca ST53 Infection

Xf damage to infected host plants varies from leaf scorch to partial desiccation to plant death. Once the bacterium entered the xylem-vessels plant system, it tries to invade all the lumina, mostly adhering to vessel wall surfaces and floating in the sap stream [49]. Bacteria reach a high concentration in xylem vessels [169], eliciting a *quorum sensing*: a communicating system among bacteria that reply to increased density by producing signal molecules [170,171,172] inducing a biofilm production. Xf reacts to quorum sensing producing a great mass of biofilm [50,173,174,175,176,177] that embeds the bacteria and plugs the vessel. The occlusion unpair the xylem-vessel water transport performances. Plant organs distal to the plug can no longer receive the water they need if intense and sudden evapotranspiration stress occurs [178].

**Figure 11:**
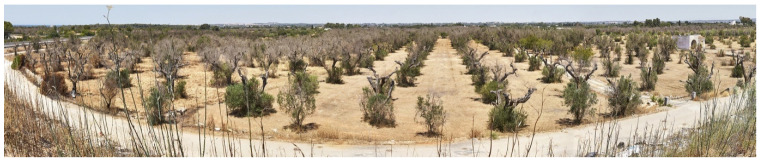
Panoramic view of an olive orchard *Xylella*-infected near Gallipoli (Apulia, Italy).

**Figure 12:**
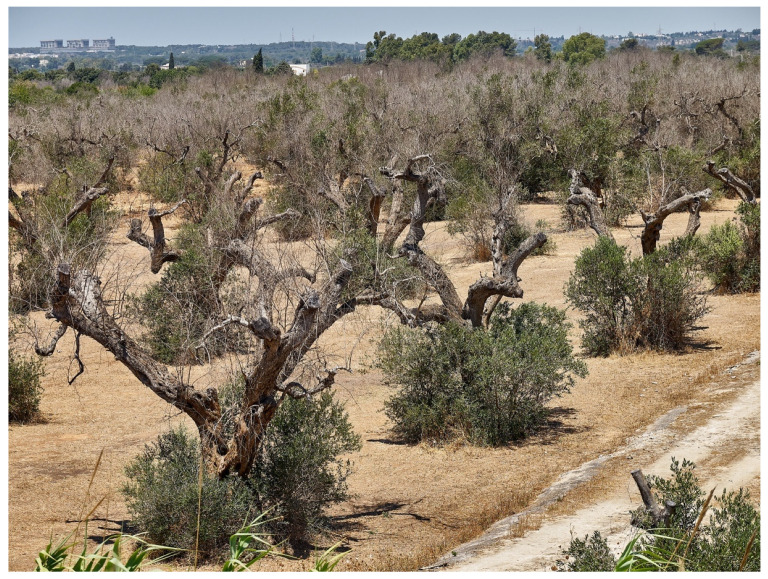
*Xylella*-infected disease olive trees in Salento with canopy drying by OQDS.

During intense and sudden evapotranspiration stressing event for hot-dry summer or cold-dry winter, the plant’s organs distal to the occlusion wilt and wither because they do not get water enough to survive [178,179,180,181] (Figure 11). The plant’s organs suffer abrupt desiccation due to environmental and unpredictable events (Figure 12); for this reason, “Disseccamento Rapido” and “Quick Decline” terms conjoin into the description of *Xylella*-associated lethal damage.

Sub-lethal damage takes place in the form of recoverable wilting, stunted stubborn-like twigs growing, miss flowering or any flowering, and other changes related to water stress that hyperspectral image analysis can perceive [182].

Another class of damage exists because of the phytosanitary measures that the sole presence of *Xylella* elicits in a Country. The Xf topics are exceedingly vast and continuously updated, requiring the EPPO and NPPO website to stay tuned with the ever-changing topic’s status of the art (https://gd.eppo.int/taxon/XYLEFA accessed on 2 August 2021).

## 5. Vector–Pathogen: Rationale Control

Here, we share the rationale to counteract the ability of vector adults to infect their food plants with Xfp53. Based on the impact of an insect-borne pathogen [183,184] vector control is the only available approach. The infectious efficiency of spittlebug vectors, the high percentage of virulent adults [185], and their mobility [36] concerning the rapid induced decline of olive suggest that *Xylella*-vectors should be considered the new key-pest of olive trees [186].

### 5.1. Control Strategy

The semi-abandoned olive orchards apparently host the highest population of Aphrophoridae, compared with the corresponding cultivated one. Moreover, the organic olive orchards are more diversified in Aphrophoridae population size compared to the other management systems. Weed control provide a significant Xf-vector population impact among olive orchards. However, insecticide applications against primary olive pests do not affect Aphrophoridae abundance [159].

**Figure 13:**
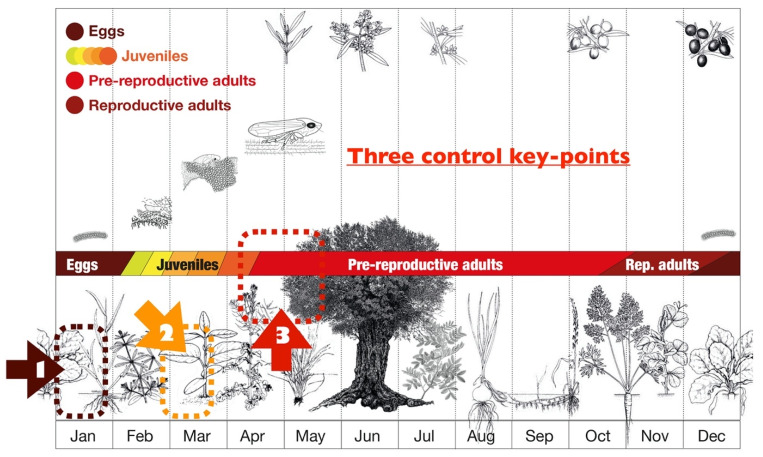
*Philaenus spumarius* management sequences: (1) control of overwintering eggs; (2) control actions against the juveniles survived; (3) control actions against survived adults in the previous control steps.

The overall management strategy consists of an integrated pest management (IPM) decision support system (DSS) [72,73,187,188,189] (Figure 13) based on quantitative sampling and vector survivor analysis with preventive and protective intents.

The model [72,73] includes three control steps, each composed of one or a few actions, targeting to reduce the vectors population size and the number of adult infecting *Xylella*-free plants. The first control step targets eggs, while the second decimates the juvenile vectors on the orchard spontaneous plants/herbs [190], and the third limits the infections up to one maximum per adult vector. The first control step relies on physical control action(s), the second on a combination of physical plus synthetics or not chemical formulates distribution, and the third on the chemical distribution of a synthetic insecticide capable of bi-directional translocation.

### 5.2. Control Step Sequences

Current management measures focus on the mechanical and chemical control of eggs and juveniles on weeds [186,191] in early spring, and later on the insecticidal application against the mass-spreading adults. The effective and fast control of adult vectors is crucial to prevent or at least mitigate the bacteria acquisition [192], avoiding the subsequent spreading of the infection and eventual damage. Modern non-conventional neonicotinoids tested [146,192] on several vector stages and field trials effectively controlled *P. spumarius*. This efficacy also originates from a quick feeding cessation followed by vector mortality resulting in low bacterial infection [72,73], higher yield and a healthier plantation in comparison to the untreated.

#### 5.2.1. First Step: Details

The first control step acts versus the population at its peak that corresponds to the overwintering eggs. Unfortunately, eggs are uncountable in the field [186] and cold-resistant [85,108,113]. The control action(s) in the first step consist(s) of one or a few winter light-tillage(s) to disrupt the sites of egg-laying, putting the naiades in trouble at born. Generally, winter tillage is a well-integrated and suggested practice necessary for water preservation [193]. It is critical to choose the tillage timing to counteract vector newborns. Tillage impact is nihil to moderate and strongly suggested in olive farming.

#### 5.2.2. Second Step: Details

Second control step acts versus the juveniles survived at the winter light-tillage that lives on spontaneous herbs array in olive orchards. Juvenile’s peak at the time of first/second nymphs corresponds to fourth and fifth juvenile instars [130]. At that time, mid-April in Southern Italy, sampling reveals that the juvenile population comprises instars from the second to the fifth [130]. However, the relevant datum is the maximum absolute number of countable individuals per unit area. *Ex-ante* and *ex-post* quantitative sampling also measure the control action efficacy versus juveniles, giving the estimation of surviving adults [130]. The impact of the quantitative sampling is null to moderate, being strongly suggested in olive farming to manage spontaneous herbs in olive farming.

Physical vector control targets nymph fourth and fifth, revealing feasible and effective [73]. However, it elicited the concern of organic management stakeholder because of the possible disturbance to soil integrity.

#### 5.2.3. Third Step: Details

It is impossible to kill all vector adult from a wide area consistently managed with olive orchards. Vectors damage does not consist in feeding acts but in the number of transmissions that each vector inflicts on the same or different target plants. The attitude to spread a pathogen among different plants [194] is the main “multiplier”, causing extensive damage and pathogen invasion. The pathogen will trigger the final damage aggressively, much more than the sole vector activity.

The first transmission to a plant is commonly called “infection”, and it is discriminating from the “infective process” occurring with the subsequent plant tissues pathogen multiplication [72,73]. The infection only changes the status of the plant from healthy to infect. The third control step kills the adults who survived the previous control actions. However, the third step could act unfruitfully as “vector control” or effectively as “infection control”, depending on the timing of the chemical control action.

In case the insecticide distribution precedes the vectors eclosion, two opportunities occur: (1) the vector feeds on a treated olive tree and dies independently on the plant status (infected/not infected); (2) the vector feeds on the non-treated infected olive tree and acquires Xfp53.

The third control step acts versus adult to impede them from feeding on the olive trees more than once [72,73], thus limiting the number of infections at the number of first feeding events. The third control step tailors the IPM strategy as:

– Preventive, to impede all the future infections from the vector because the insect dies on the tree during Xfp53 acquisition;
– Protective, to limit the vector action to one infection; vector dies on the just infected twig and impeding the infection of other olive trees or the repeated transmissions on the same plant.

#### 5.2.4. Control Steps: More about

The unwanted introduction of Xfp53 in Italy initiated an invasion after a reasonable period of latency. The date of Xfp53 entrance in Italy is uncertain; estimations also formulate in connection with the findings of *Aleurocanthus spiniferus* Quaintance 1903 [194,195,196] in the same area suggest an entrance from 2000 to 2003. Many coffee ornamental plants [21] entered Europe and Italy. Subsequently, the coffee plant was shown to carry the bacterium with not lethal and not specific leaf scorch damage. In the Gallipoli area (Lecce province) coffee plants, and other unspecified infected ornamentals from Central American farms, can survive and prosper outdoor all year round. In the same environment, free-living adult vectors were able to visit those plant, acquiring Xfp53. The acquisition resulted in indigenous plants infection, triggering the Italian invasion.

Invasion is still in the act and will end in an unpredictable time. The main argument to consider is “The Thin Red Line” (https://en.wikipedia.org/wiki/The_Thin_Red_Line_(novel) access on 22 July 2021) of invasion that corresponds to the border of the infected area and not to the limit of symptomatic plants. Any action targeted on vectors should be enforced ahead of “The Thin Red Line”, both in the asymptomatic and symptomatic area, to be successful. Acting only in the symptomatic belt behind the infection line is a lose–lose strategy that will negatively affect the invasion control [197].

Unfortunately, “The Thin Red Line” is invisible to the naked eye and almost impossible to detect by instrumental analysis. Detection of the first few millimeters of a twig infected by a vector could be helpful but complex because it is included in identical hundreds or thousands of olive twigs. The success in mitigating and stopping the invasion goes through deploying an IPM strategy for infection control in the area preceding the symptomatic one [198,199] or before “The Thin Red Line”. Any action targeting the invasion will work and be eventually successful only if applied at least far ahead of the invisible “Thin Red Line”.

Another reason to impose control in not-yet-infected areas is preventing the infection foci due to the occasional and passive dispersion of viruliferous adults. A critical factor in Xfp53 invasion containment is to lower the probability that resident-vectors will acquire from infected twigs previously fed by viruliferous vectors. The same activities for the vector population control that works in endemic and symptomatic belts work better in areas devoid of the bacterium because the number of the vectors is exceedingly low [72,73].

The infection control steps may use chemical or biological control actions [72,73]. One or a sequence of few distributions of synthetic insecticide by tree injection will contain the infections, depending on the size of the adult target population. The infection control action could also consist of vector biocontrol via antagonists’ inundation, e.g., Reduviidae [73,186]. Delaying the adults’ control allows multiple infections per vector and demonstrates the difference between ineffective and effective infection control.

### 5.3. Quantitative Control Approach

Accepting that the critical event in the pathogen invasion is the infection, we consider the key parameter the ratio between the number of possible infections and the number of susceptible plants. As in Fierro [72] and Bucci [200] discussion, any control actions proposed must concern the management of several vectors because capable of inflicting many infections. The quantitative approach is the only available (=effective) to plan, execute, harmonize, and verify a proper IPM strategy [201].

### 5.4. Symptomatic Plants Uprooting

The uprooting and extirpation of OQDS infected-asymptomatic or symptomatic plants is not an effective action because those plants have been acting as bacterium reservoirs for one or more years (3–4) at least [202]. The limit of 50–100 m ray for uprooting/extirpating [168] around an infected tree is ineffective because the active vector dispersion per year exceeds some hundred meters [36]. The vectors acquire the pathogen from the infected plant and transmit it to other plants in the ray of 200–400 m before that focal plant revealed to be infected. The idea to uproot/extirpate diseased or asymptomatic plants only helps to mitigate the pathogen invasion. However, the accurate vector control “locks” the pathogen into the infected plant—not only symptomatic—can act better than infected plant uprooting, preventing the spread of Xfp53.

### 5.5. Vector Census

The juvenile-vector population size knowledge is crucial in establishing an effective control strategy whereby choosing action thresholds, tuning control action intensity, and estimating control efficacy [73]. The first experience on spittle quantitative sampling, by direct in-field scrutiny, revealed an expensive approach in time and workforce [185]. Moreover, the population size gathered data are not congruous among pre-imaginal instar. Divergence in observation could be due to the small size of the early juvenile instar, the spittle inconsistency, and the inherent difficulty collecting all from a growing herb volume (0.5–0.8 m^3^/m^2^).

Quantitative sampling can forecast the timing and tune the control actions intensity [130]; both points are crucial for the overall control efficacy. Without accurate timing and the proper control intensity, the IPM will provoke either side effects or results ineffective. Quantitative sampling verifies the expected results, control actions efficacy, and confirms the side effect level.

Several spittlebugs share the behavior and the common name. They can infest uncultured fields [148], amassing an exceedingly high population and difficult to manage because of their protected lifestyle. Ps control was marginal until the demonstration of its ability to transmit the Xfp53. Moreover, the percentage of Ps [151,186] over the total foamy-mass number is almost unpredictable and the missing of a selective control means, forced to control the entire spittlebugs population either as juveniles or adults. Furthermore, control of spittlebug juveniles is strongly preferred because they have a negligible ability to transmit Xfp53 [191].

AquaSamPling (ASP) is an appropriate area-wide quantitative sampling technique purposely tailored for spittlebugs juvenile census [130,203,204]. The technique is based on the (micro) habitat plant-unit removal [205] and following in-liquid insect extraction to get complete census counting [130]. The juveniles sampling technique used in EFSA accounts [124] and sweeping net [206,207] are not considered because phenetic and non-quantitative. They return a count of individual neither actual nor valuable for the vector control. Furthermore, Kretzschmar [208] and Pedigo [209] consider the sweeping an underestimating sampling method for immatures. The need for a quantitative evaluation of the target population (Aphrophoridae, until now) exists because the control targets are the Aphrophoridae as vectors and not as conventionally damaging pests [130]. In the case of conventionally dangerous pest [62,210,211], even a non-quantitative pest population census method works because the need is to correlate the number of individuals (e.g., per trap) with an (economic/action) threshold. Such a correlation considers the damage rather than population census.

In vectors, the main parameter is the ratio between the number of vectors and the number of susceptible hosts [190]. The vector control aims to minimize the absolute number below a threshold during juvenile instars to protect the susceptible host plants and prevent them from infections by the survived adults [72,73]. The actual population census by ASP shows the need to manage a population estimate to range from one to one hundred million vectors per hectare [130] to gain a possible coexistence with Xfp53 in Italy.

### 5.6. Actual Engagement

Several xylem sap-feeders Hemipteran are or will be candidate key-pests for the Mediterranean inasmuch vectors of Xfp53 in Apulia. Nonspecific vectors could transmit Xf [38]; local xylem-feeders may also transmit the bacterium when this one enters a new biogeographical region [212]. Therefore, the study’s main aim in not-yet-invaded countries is to assess the guild of possible local Xf vectors to improve the surveillance and build an effective DSS IPM control strategy in time [201].

OQDS affects millions of olive trees, threatening three-quarters of the world’s olive oil production [213]. Recent estimates quantified the desiccation of more than 6,500,000 olive trees [214], and the infected area in Southern Italy continues to expand.

Schneider et al. [213] argue that both producers and consumers suffer the implications of Xfp invasion. Most of the impacts fall on consumers as higher olive oil prices, and reservoir shortening. The next dispersion of the pathogen would exacerbate the scenario causing significant agricultural, environmental, social [167], and health [215] impacts.

OQDS imposes a reduction in the supply of ecosystem services derived from olive orchards of 30–34% and a decrease in associated biodiversity of 28%, in addition to the impacts on productivity and the entire olive oil supply chain [216].

The current economic impact is conspicuous, and future projections do not promise an excellent productive scenario. The economic benefit derived from reducing the spread in *Xylella*-free areas and implementing mitigation measures in affected areas could guarantee a reduction of the disease impact ranged from 41% to 91% [71].
